# Accelerating innovative publishing in taxonomy and systematics: 250 issues of ZooKeys

**DOI:** 10.3897/zookeys.251.4516

**Published:** 2012-12-18

**Authors:** Terry Erwin, Lyubomir Penev, Pavel Stoev, Teodor Georgiev

**Affiliations:** 1 Smithsonian Institution, Washington, DC, USA; 2 Bulgarian Academy of Sciences & Pensoft Publishers, Sofia, Bulgaria; 3 Pensoft Publishers, Sofia, Bulgaria

‘*Every good product I’ve ever seen is because a group of people cared deeply about making something wonderful that they and their friends wanted. They wanted to use it themselves*’

Steve Jobs

On 13^th^ of December ZooKeys published issue 250, the 76-page monograph “Introduction of the *Exocelina ekari*-group with descriptions of 22 new species from New Guinea (Coleoptera, Dytiscidae, Copelatinae)” by Shaverdo et al. (2012). Likewise, this semiquincentennial issue is a milestone for the editorial team to take a look back and evaluate the journal’s progress in the past year.

The year 2012 has been important for the journal and the entire zoological community in many aspects. On the 4th of September, the International Commission on Zoological Nomenclature (ICZN) passed an amendment that considers a publication in a digital scientific journal ‘legitimate’ if meeting archiving criteria and the publication is registered at the ICZN’s official online registry, ZooBank. Pensoft was among the first supporters of the open-access idea, and one of the most active advocates of the e-only publishing of taxonomic data. Taking this into account and as recognition of Pensoft’s active role in promotion of taxonomy, the Commission decided to announce the Amendment of the Code in ZooKeys (ICZN 2012). This revolutionary change in ICZN publication rules will undoubtedly speed the process of publishing biodiversity information and improve access to this information.

As a result of a fruitful collaboration, The Encyclopedia of Life (EOL) and ZooKeys on February 10th, 2012 announced a new joint project (EOASP) aimed at increasing the flow of new species descriptions from scientists in developing countries into the Encyclopedia of Life and promoting the open access publishing model in taxonomy. Another goal of this initiative was to support and educate the next generation of taxonomists in open science principles and to motivate publishers to modernize their publishing models and workflows to fit the changing needs of scientists and researchers around the world. Up to now, the EOASP project has supported 22 articles, in which 32 species, 7 genera and 1 family were described as new to sciences, and several more taxa were re-described.

The EOASP project was preceeded by another joint initiative with EOL called “Fabulous New Species collection” launched in January 2012. Its main aim was to bring together and promulgate the scientifically notable new taxa described every year in ZooKeys and other Pensoft’s journals and simultaneously registered in EOL. A short annotation written in a popular language explains why the new species is interesting in an attempt to draw the attention of the general public and the world mass media to it.

In 2012, a new pilot project for establishing the Online Identification Key (OIK) as a new type of scientific article that is a derivative of the Data Paper was put forward by ZooKeys. The publication of an online key in the form of a scholarly article is a pragmatic compromise between the dynamic structure of the internet and the static character of scientific articles. The authors of the keys will be able to continuously update their products, to the benefit of users. At the same time, the users will have available a citation mechanism for the online key, identical to that used for any other scientific article, to properly credit its author(s). The model is illustrated by an exemplar paper describing a new software platform for creating online keys, MOSCHweb (Cerretti et al. 2012). The paper describes the main features of an interactive key to the Euro-Asiatic genera of tachinid flies implemented as an original web application and discusses briefly the advantages of these tools for both biologists and general users.

The year 2012 will be remembered also with the ZooKeys special issue “No specimen left behind: mass digitization of natural history collections” initiated by the Natural History Museum in London. The editors Vince Smith and Vladimir Blagoderov brought together 18 papers by 81 authors to look at progress and prospects for mass digitizing of entire natural history collections. The compendium examines recent advances in imaging systems and data gathering techniques, combined with more collaborative approaches to digitization. Examples of research covered by the articles include a description of efforts to digitize 30 million plant, insect and vertebrate specimens at NCB Naturalis in the Netherlands; new scanning and telemicroscopy solutions to digitize the millions of pinned insect specimens held in the Australian National Insect Collection and its European and North American counterparts; and, new data portals providing central access to millions of biological specimens across Europe, etc.

The discovery of any organism in the world matters equally, be it a minute insect or a mammal. Several newly discovered animals published in ZooKeys at the verge of 2011/2012 and in 2012 were of immense interest and attracted the attention of the global society. These include, among others, the world’s smallest tetrapode, the largest wasp, and the leggiest animal, as well as a new fanged dinosaur from Africaand new family of cave spiders from the USA. In December 2011, ZooKeys announced the publication of the world’s smallest vertebrates – the New Guinean frogs *Paedophryne dekot* and *Paedophryne verrucosa*, which average length rarely exceeds 8–9 mm (Kraus 2011). The press release associated with the publication grabbed the interest of the world’s media and shortly reached 44, 000 views – an absolute record for ZooKeys – in Eurekalert! alone. Shortly after the publication even smaller species of the same genus were found in New Guinea and published in PLoS One (Rittmeyer et al. 2012).

**Figure 1.**
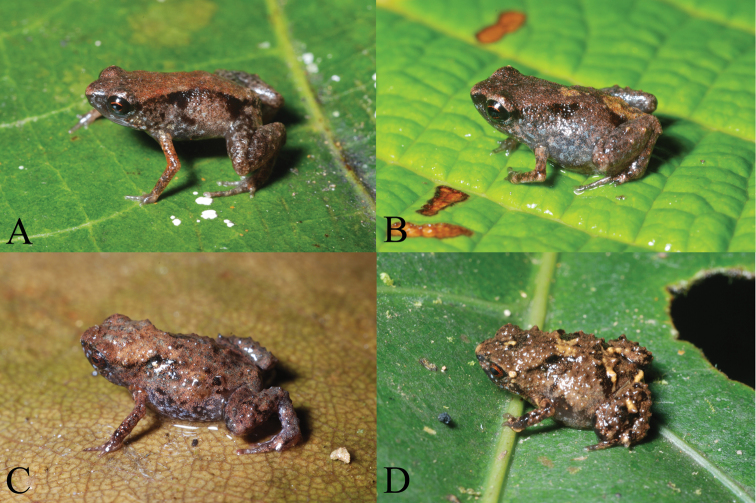
Portraits in life of *Paedophryne dekot* (A, B) and *Paedophryne verrucosa* (C, D). Photos: Fred Kraus, 2011. ZooKeys: doi: 10.3897/zookeys.154.1963

A new species of plant-eating dinosaur, named *Pegomastax africanus*, or “thick jaw from Africa”, was described in ZooKeys by Professor Paul Sereno of the University of Chicago in October 2012 (Sereno 2012). The new species had a short, parrot-shaped beak up front, a pair of stabbing canines and tall teeth tucked behind for slicing plants. The dinosaur was of cat size and lived some 200 million years ago in Africa. It was placed by its finder in the heterodontosaurs or “different toothed reptiles,” that were among the first dinosaurs to spread across the planet. The discovery was featured by all the world’s leading news media, e.g.,: **BBC**: Dwarf ‘vampire dinosar was a plant eater, **CNN**: Scientist describes fruit-loving, housecat-sized dino, **National Geographic**: New fanged dwarf dinosaur found, “Would be nice pet”, **New York Times**: Bizarre species of miniature dinosaur identified, **The Guardian**: “Fanged vampire parrot” identified as a new species of dinosaur, **Scientific American**: Diminutive dinosaur bore beak, bristles and fangs, **USA Today**: Fanged dinosaur feasted on fruit, **Time**: A parrot-headed, big-fanged, porcupine dinosaur.

**Figure 2.**
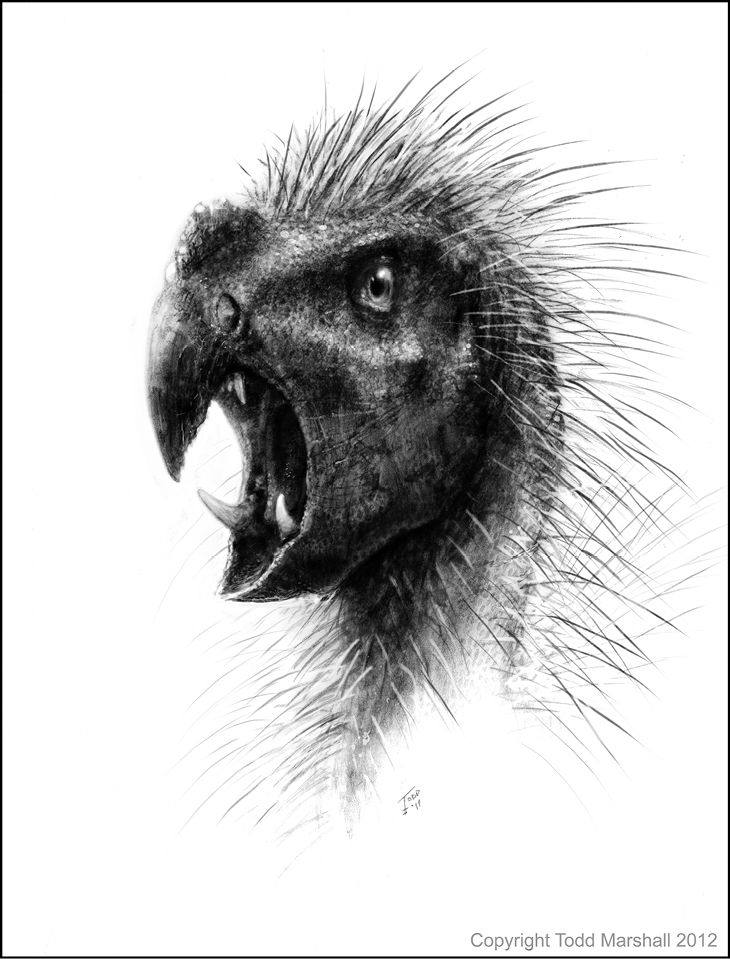
The head of *Pegomastax africanus*. Reconstruction by Todd Marshall, 2012.

An unusually large wasp species, named by the authors *Megalara garuda*, was discovered during an expedition to the Indonesian island of Sulawesi (Kimsey and Ohl 2012). With a body size of 32–34 mm and fearful jaws in males, the new species differs from all known related digger wasps, so much so that it was placed in a new genus of its own, *Megalara*.

**Figure:**
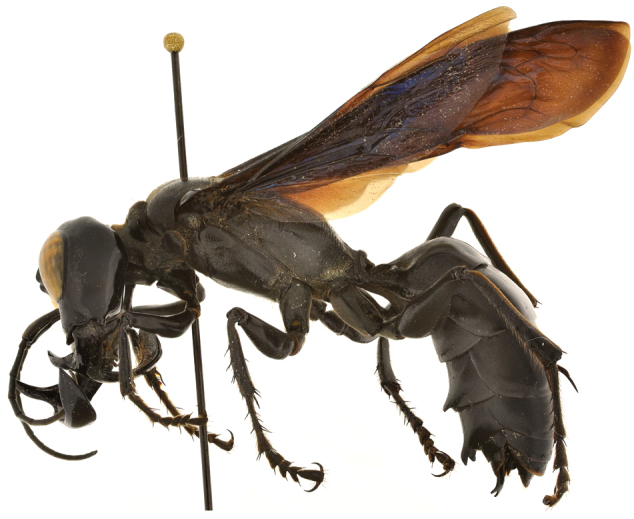
**Figure 3.** Habitus of *Megalara garuda*. Photo: Kimsey and Ohl 2012. ZooKeys: doi: 10.3897/zookeys.177.2475

A team of citizen scientists from the Western Cave Conservancy and arachnologists from the California Academy of Sciences found in caves in southwest Oregon, USA, a new, previously unknown family of spiders, named Trogloraptoridae (after the genus *Trogloraptor* or “cave robber”) that was published in ZooKeys in August 2012 (Griswold et al. 2012). The discovery has turned into the most visited ZooKeys paper ever, with more than 34,000 at the time of publishing this Editorial. The paper was also featured by CNN, The New York Times, Scientific American and on BBC Today, among many other media. The new species is named for its cave home and spectacular, elongate claws.

International recognition was received also on the discovery of *Kollasmosoma sentum*, a new tiny parasitic braconid wasp with unusual behavior (Gómez Durán and Achterberg 2011). It was selected by the International Institute for Species Exploration and included in their 2011 top 10 list of world’s most interesting new species.

**Figure 4.**
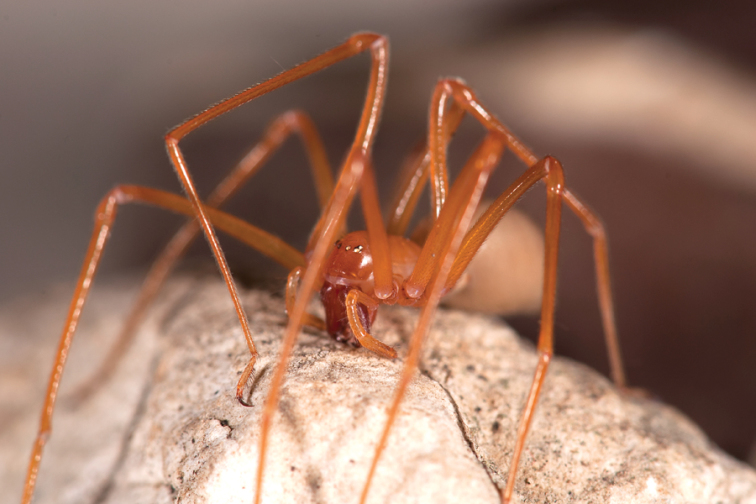
Habitus of live *Trogloraptor marchingtoni*. Photo: Griswold et al. 2012. ZooKeys: doi: 10.3897/zookeys.215.3547

Marek et al. (2012) re-described the leggiest animal in the world, the millipede *lllacme plenipes*, re-discovered several years ago in California. The females have up to an astounding 750 legs, outclassing the males who only have a maximum leg count of 562. Not only is this species the leggiest animal known on the planet, it also has surprising anatomical features: body hairs that produce silk, a jagged and scaly translucent exoskeleton, and comparatively massive (given its diminutive size) antennae that are used to feel its way through the dark because it lacks eyes. The video associated with the publication enjoyed more than 46,500 views in YouTube within only a month.

**Figure 5.**
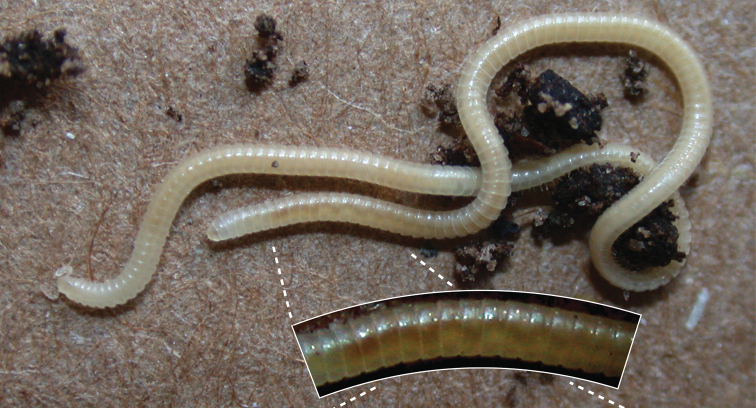
Habitus of live *Illacme plenipes* ♀ with 170 segments and 662 legs. Photo: Marek et al. 2012. ZooKeys: doi: 10.3897/zookeys.241.3831

## Four and a half years ZooKeys

**Figure 6.**
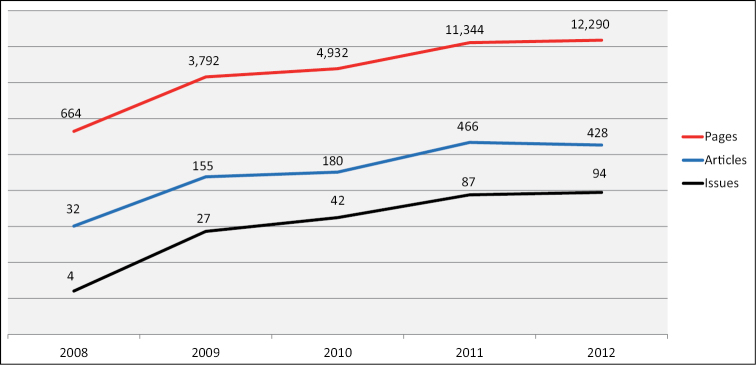
Total number of published pages, articles and issues for the period 2008–2012.

From the 4^th^ of July 2008 until the 10^th^ of December 2012 the number of published articles and monographs has reached 1,245 or 32,416 pages overall. The number of published pages has grown from 11,344 in 2011 to 12,292 in 2012 (Fig. 6). Based on the analysis of 40 randomly selected papers published in 2012, the average publication time (from submission to publication) is 95 days. The average time from submission to acceptance is 72 days, and from acceptance to publication – 23 day.

Altogether, 2,904 new species-group, 233 new genus-group and 19 new family-group taxa have been published in the journal since its launch. Another 69 are currently in press (being registered in ZooBank) and will probably be published by the end of 2012 or early 2013. This makes 2,973 new taxa in total.

The top 10 most accessed ZooKeys papers through the 10^th^ of December 2012 are listed in Table 1. The remarkable discovery of the new spider family Trogloraptoridae by Griswold et al. (2012) published in August is taking unquestioningly the lead with 33,492 page views, or approximately 20,000 views more than the Bouchard et al.’s monograph (2011) “Family-Group names in Coleoptera (Insecta)” taking a second place. The total number of views of the top 10 most accessed ZooKeys articles exceeds 135,000, which along with the fact that half of them were published in 2012, speaks enough of the continuous importance of the published content and the increasing interest in taxonomic community in the ZooKeys publications as a whole.

**Table 1.** Top ten most viewed articles of ZooKeys (according to the ZooKeys website counter accessed on the 10^th^ of December 2012).

Article	Page views
Griswold et al. 2012 – An extraordinary new family of spiders from caves in the Pacific Northwest (Araneae, Trogloraptoridae, new family)	34,003
Bouchard et al. 2011 – Family-Group names in Coleoptera (Insecta)	13,619
Winterton et al. 2012 – A charismatic new species of green lacewing discovered in Malaysia (Neuroptera, Chrysopidae): the confluence of citizen scientist, online image database and cybertaxonomy	12,776
Sereno 2012 – Taxonomy, morphology, masticatory function and phylogeny of heterodontosaurid dinosaurs	12,724
Penev et al. 2009 – Data publication and dissemination of interactive keys under the open access model	12,307
Kraus 2011 – At the lower size limit for tetrapods, two new species of the miniaturized frog genus Paedophryne (Anura, Microhylidae)	12,139
Hagedorn et al. 2011 – Creative Commons licenses and the non-commercial condition: Implications for the re-use of biodiversity information	10,885
Kimsey and Ohl 2012 – Megalara garuda, a new genus and species of larrine wasps from Indonesia (Larrinae, Crabronidae, Hymenoptera)	9,698
Sereno and Larsson 2009 – Cretaceous Crocodyliforms from the Sahara	9,392
Baldwin et al. 2011 – Seven new species within western Atlantic Starksia atlantica, S. lepicoelia, and S. sluiteri (Teleostei, Labrisomidae), with comments on congruence of DNA barcodes and species	8,087
Total	135,119

In 2012, ZooKeys continued to popularize the published research through press releases, which in most cases were broadly mirrored by the global science media and increased the visibility of the journal on the global scene. A list of the top 10 most accessed posted through EurekAlert! press releases of ZooKeys articles is given in Table 2. Top two places are taken by press releases that were viewed by more than 44,000 science media and individual journalists, third position lying far below that number attracting the attention of ‘only’ around 16,000 communication experts. The top three most accessed press releases logically find place also in the “top 10” list of most viewed papers on the ZooKeys website.

**Table 2.** Top 10 most accessed press releases of ZooKeys articles posted through EurekAlert! (from the EurekAlert! counter) for the period 1 December 2011 – 10 December 2012. The counter registers only the downloads from EurekAlert! mostly by science media and journalists. The actual number of readers may actually be a much higher than this number.

Title	Author/s and year of publication of the original article	Date posted	Page views since posted
*Megalara garuda*: the King of Wasps: A new, giant wasp comes from Indonesia	Kimsey and Ohl 2012	23-Mar-2012	44,669
World’s smallest frogs discovered in New Guinea	Kraus 2011	12-Dec-2011	44,247
Spider version of Bigfoot emerges from caves in the Pacific Northwest	Griswold et al. 2012	17-Aug-2012	16,361
The Auburn Tiger trapdoor spider -- a new species discovered from a college town backyard	Bond et al. 2012	8-May-2012	5,012
Millipede border control better than ours	Mesibov 2011	23-Dec-2011	4,592
9 colorful and endangered tree-dwelling tarantulas discovered in Brazil	Bertani 2012	30-Oct-2012	4,252
DNA barcoding verified the discovery of a highly disconnected crane fly species	Pilipenko et al. 2012	18-May-2012	4,205
Scorpio rising: An elusive new scorpion species from California lives underground	Webber et al. 2012	23-Mar-2012	3,247
Italian vineyards invaded from North America by new species of leafminer	Nieukerken et al. 2012	23-Feb-2012	3,160
A new, beautifully colored lizard discovered in the Peruvian Andes: Named ‘mountain dweller’, it is the highest-altitude living member of its genus	Chávez and Vásquez 2012	17-Feb-2012	3,093

In conclusion, Pensoft Publishers’ flagship taxonomic journal ZooKeys continues to experience growth in 2012. Furthermore, ZooKeys continues to evolve its editorial workflow, constantly implementing new and improved publishing and dissemination technologies, thus always being on point for digital biodiversity science. This is backed up by the fact that the Impact Factor of ZooKeys has increased from 0.517 to 0.897 in 2011, and is expected to continue growing due to the high quality and great visibility of the published content. We are deeply indebted to all our authors, reviewers, subject editors, and readers, and journalistic followers without whose support ZooKeys would not have become such a successful journal! We also thank Rich Pyle for providing information on the new taxa registered in ZooBank.

## References

[B1] Baldwin CC Castillo CI Weigt LA Victor BC (2011) Seven new species within western Atlantic *Starksia atlantica*, *S. lepicoelia*, and *S. sluiteri* (Teleostei, Labrisomidae), with comments on congruence of DNA barcodes and species. ZooKeys 79: 21-72 doi: 10.3897/zookeys.79.1045 21594143 10.3897/zookeys.79.1045 PMC3088046

[B2] Bertani R (2012) Revision, cladistic analysis and biogeography of *Typhochlaena* C. L. Koch, 1850, *Pachistopelma* Pocock, 1901 and *Iridopelma* Pocock, 1901 (Araneae, Theraphosidae, Aviculariinae). ZooKeys 230: 1-94 doi: 10.3897/zookeys.230.3500 23166476 10.3897/zookeys.230.3500 PMC3494022

[B3] Blagoderov V Smith VS (2012) Bringing collections out of the dark. In: Blagoderov V Smith VS (Ed). No specimen left behind: mass digitization of natural history collections.ZooKeys 209: 1–6 doi: 10.3897/zookeys.209.3699﻿﻿﻿﻿﻿﻿﻿﻿﻿﻿﻿﻿﻿﻿﻿﻿﻿﻿﻿﻿﻿﻿﻿﻿﻿﻿﻿﻿﻿﻿﻿﻿﻿﻿﻿﻿﻿﻿﻿﻿﻿﻿﻿﻿﻿﻿﻿﻿﻿﻿﻿﻿﻿﻿﻿﻿﻿﻿﻿﻿﻿﻿﻿﻿ 22859874 10.3897/zookeys.209.3699 PMC3406462

[B4] Bond JE Hamilton CA Garrison NL Ray CH (2012) Phylogenetic reconsideration of *Myrmekiaphila* systematics with a description of the new trapdoor spider species *Myrmekiaphila tigris* (Araneae, Mygalomorphae, Cyrtaucheniidae, Euctenizinae) from Auburn, Alabama. ZooKeys 190: 95-109 doi: 10.3897/zookeys.190.3011 22639533 10.3897/zookeys.190.3011 PMC3349069

[B5] Bouchard P Bousquet Y Davies AE Alonso-Zarazaga MA Lawrence JF Lyal CHC Newton AF Reid CAM Schmitt M Ślipiński SA Smith ABT (2011) Family-group names in Coleoptera (Insecta). ZooKeys 88: 1-972 doi: 10.3897/zookeys.88.807 21594053 10.3897/zookeys.88.807 PMC3088472

[B6] Cerretti P Tschorsnig H-P Lopresti M Di Giovanni F (2012) MOSCHweb — a matrix-based interactive key to the genera of the Palaearctic Tachinidae (Insecta, Diptera). ZooKeys 205: 5-18 doi: 10.3897/zookeys.205.3409 22792031 10.3897/zookeys.205.3409 PMC3391727

[B7] Chávez G Vásquez D (2012) A new species of Andean semiaquatic lizard of the genus *Potamites* (Sauria, Gymnophtalmidae) from southern Peru. ZooKeys 168: 31-44 doi: 10.3897/zookeys.168.2048﻿﻿﻿﻿﻿﻿﻿﻿﻿﻿﻿﻿﻿﻿﻿﻿﻿﻿﻿﻿﻿﻿﻿﻿﻿﻿﻿﻿﻿﻿﻿﻿﻿﻿﻿﻿﻿﻿﻿﻿﻿﻿﻿﻿﻿﻿﻿﻿﻿﻿﻿﻿﻿﻿﻿﻿﻿﻿﻿﻿﻿﻿﻿﻿ 22423189 10.3897/zookeys.168.2048 PMC3293442

[B8] Gómez Durán J-M van Achterberg C (2011) Oviposition behaviour of four ant parasitoids (Hymenoptera, Braconidae, Euphorinae, Neoneurini and Ichneumonidae, Hybrizontinae), with the description of three new European species. ZooKeys 125: 59-106 doi: 10.3897/zookeys.125.1754 21998538 10.3897/zookeys.125.1754 PMC3185369

[B9] Griswold CE Audisio T Ledford JM (2012) An extraordinary new family of spiders from caves in the Pacific Northwest (Araneae, Trogloraptoridae, new family). ZooKeys 215: 77-102 doi: 10.3897/zookeys.215.3547﻿﻿﻿﻿﻿﻿﻿﻿﻿﻿﻿﻿﻿﻿﻿﻿﻿﻿﻿﻿﻿﻿﻿﻿﻿﻿﻿﻿﻿﻿﻿﻿﻿﻿﻿﻿﻿﻿﻿﻿﻿﻿﻿﻿﻿﻿﻿﻿﻿﻿﻿﻿﻿﻿﻿﻿﻿﻿﻿﻿﻿﻿﻿﻿ 22936872 10.3897/zookeys.215.3547 PMC3428790

[B10] Hagedorn G Mietchen D Morris RA Agosti D Penev L Berendsohn WG Hobern D (2011) Creative Commons licenses and the non-commercial condition: Implications for the re-use of biodiversity information. In: Smith V Penev L (Eds). e-Infrastructures for data publishing in biodiversity science.ZooKeys 150: 127–149 doi: 10.3897/zookeys.150.2189 22207810 10.3897/zookeys.150.2189 PMC3234435

[B11] International Commission on Zoological Nomenclature (2012) Amendment of Articles 8, 9, 10, 21 and 78 of the International Code of Zoological Nomenclature to expand and refine methods of publication. ZooKeys 219: 1-10 doi: 10.3897/zookeys.219.3944 22977348 10.3897/zookeys.219.3994 PMC3433695

[B12] Kimsey LS Ohl M (2012) *Megalara garuda*, a new genus and species of larrine wasps from Indonesia (Larrinae, Crabronidae, Hymenoptera). ZooKeys 177: 49-57 doi: 10.3897/zookeys.177.2475 22532785 10.3897/zookeys.177.2475 PMC3317617

[B13] Kraus F (2011) At the lower size limit for tetrapods, two new species of the miniaturized frog genus *Paedophryne* (Anura, Microhylidae). ZooKeys 154: 71-88 doi: 10.3897/zookeys.154.1963 22287916 10.3897/zookeys.154.1963 PMC3238040

[B14] Marek PE Shear WA Bond JE (2012) A redescription of the leggiest animal, the millipede *Illacme plenipes*, with notes on its natural history and biogeography (Diplopoda, Siphonophorida, Siphonorhinidae). ZooKeys 241: 77-112 doi: 10.3897/zookeys.241.3831﻿﻿﻿﻿﻿﻿﻿﻿﻿﻿﻿﻿﻿﻿﻿﻿﻿﻿﻿﻿﻿﻿﻿﻿﻿﻿﻿﻿﻿﻿﻿﻿﻿﻿﻿﻿﻿﻿﻿﻿﻿﻿﻿﻿﻿﻿﻿﻿﻿﻿﻿﻿﻿﻿﻿﻿﻿﻿﻿﻿﻿﻿﻿﻿ 10.3897/zookeys.241.3831 PMC3559107 23372415

[B15] Mesibov R (2011) A remarkable case of mosaic parapatry in millipedes. In: Mesibov R Short M (Eds). Proceedings of the 15th International Congress of Myriapodology, 18-22 July 2011, Brisbane, Australia.ZooKeys 156: 71–84 doi: 10.3897/zookeys.156.1893 22303096 10.3897/zookeys.156.1893 PMC3253572

[B16] Nieukerken EJ van Wagner DL Baldessari M Mazzon L Angeli G Girolami V Duso C Doorenweerd C (2012) *Antispila oinophylla* new species (Lepidoptera, Heliozelidae), a new North American grapevine leafminer invading Italian vineyards: taxonomy, DNA barcodes and life cycle. ZooKeys 170: 29-77 doi: 10.3897/zookeys.170.2617 22408380 10.3897/zookeys.170.2617 PMC3288679

[B17] Penev L Sharkey M Erwin T van Noort S Buffington M Seltmann K Johnson N Taylor M Thompson FC Dallwitz MJ (2009) Data publication and dissemination of interactive keys under the open access model: ZooKeys working example. ZooKeys 21: 1-17 doi: 10.3897/zookeys.21.274

[B18] Penev L Cerretti P Tschorsnig H-P Lopresti M Di Giovanni F Georgiev T Stoev P (2012) Publishing online identification keys in the form of scholarly papers. ZooKeys 205: 1-3 doi: 10.3897/zookeys.205.3581 22792030 10.3897/zookeys.205.3581 PMC3391726

[B19] Pilipenko VE Salmela J Vesterinen EJ (2012) Description and DNA barcoding of *Tipula (Pterelachisus) recondita* sp. n. from the Palaearctic region (Diptera, Tipulidae). ZooKeys 192: 51-65 doi: 10.3897/zookeys.192.2364 22639539 10.3897/zookeys.192.2364 PMC3349062

[B20] Rittmeyer EN Allison A Gründler MC Thompson DK Austin CC (2012) Ecological Guild Evolution and the Discovery of the World’s Smallest Vertebrate. PLoS ONE 7(1): e29797. doi: 10.1371/journal.pone.0029797 10.1371/journal.pone.0029797 PMC3256195 22253785

[B21] Sereno PC (2012) Taxonomy, morphology, masticatory function and phylogeny of heterodontosaurid dinosaurs. ZooKeys 226: 1-225 doi: 10.3897/zookeys.226.2840 23166466 10.3897/zookeys.227.4091 PMC3487651

[B22] Sereno PC Larsson HCE (2009) Cretaceous Crocodyliforms from the Sahara. ZooKeys 28: 1-143 doi: 10.3897/zookeys.28.325

[B23] Shaverdo HV Surbakti S Hendrich L Balke M (2012) Introduction of the *Exocelina ekari*-group with descriptions of 22 new species from New Guinea (Coleoptera, Dytiscidae, Copelatinae). ZooKeys 250: 1-76 doi: 10.3897/zookeys.250.3715 10.3897/zookeys.250.3715 PMC3558971 23378803

[B24] Webber MM Graham MR Jaeger JR (2012) *Wernerius inyoensis*, an elusive new scorpion from the Inyo Mountains of California (Scorpiones, Vaejovidae). ZooKeys 177: 1-13 doi: 10.3897/zookeys.177.2562 22532782 10.3897/zookeys.177.2562 PMC3317614

[B25] Winterton SL Guek HP Brooks SJ (2012) A charismatic new species of green lacewing discovered in Malaysia (Neuroptera, Chrysopidae): the confluence of citizen scientist, online image database and cybertaxonomy. ZooKeys 214: 1-11 doi: 10.3897/zookeys.214.3220 22936863 10.3897/zookeys.214.3220 PMC3426877

